# Software-related challenges in Swedish healthcare through the lens of incident reports: A desktop study

**DOI:** 10.1177/20552076231203600

**Published:** 2023-09-20

**Authors:** Md Shafiqur Rahman Jabin, Ding Pan

**Affiliations:** 1 Department of Medicine and Optometry, Linnaeus University, Kalmar, Sweden; 2 Faculty of Health Studies, University of Bradford, Bradford, UK; 3 Faculty of Health and Life Sciences, 4180 Linnaeus University, Kalmar, Sweden

**Keywords:** Software interface, system configuration, system design, data protection legislation, software functionality, decision-support system

## Abstract

**Objective:**

To identify a subset of software issues occurring in daily Swedish healthcare practice and devise a set of local solutions to overcome the challenges.

**Methods:**

A sample of 46 incident reports was collected from one of Sweden's national incident reporting repositories, ranging from June 2019 to December 2021. The reports were first subjected to an algorithm to identify if they were health information technology-related incidents and were analysed using an existing framework, i.e., the Health Information Technology Classification System, to identify the software-related incidents. The incidents associated with software issues were then subjected to thematic analysis, in which themes were extracted and presented under the category assigned by the existing framework used.

**Results:**

Of 46 reports, 45 (with one exception) were included using the algorithm. Of 45 incidents, 31 software-related incidents were identified using the classification system. Six types of software issues were identified, including software functionality (*n* = 10), interface with other software systems or components (*n* = 10), system configuration (*n* = 7), interface with devices (*n* = 2), record migration (*n* = 1) and increased volume of transactions (*n* = 1). Each issue was further categorised into different themes; for example, software interface-related problems were grouped into ‘two patients being active in the system simultaneously’ (*n* = 6) and ‘transfer of patient information’ (*n* = 4).

**Conclusions:**

The study provided some insights into software issues and relevant consequences. A set of local solutions were devised to overcome the present challenges encountered in Swedish healthcare in their daily clinical practice. Systematic identification and characterisation of such software challenges should be a routine part of clinical practice for all major health information technology implementations.

## Introduction

Health information technology (HIT) is defined as ‘hardware or software that is used to electronically create, maintain, analyse, store and receive information, or otherwise aid in the diagnosis, cure, mitigation, treatment or prevention of disease, that is not an integral part of an implantable device or medical equipment’.^1^ Over the last three decades, HIT has been an integral part of modern medicine for improving healthcare workflow and services to be more effective and efficient.^2^ However, HIT system deployment and adoption can create considerable disruptions stemming from issues of interoperability and interfaces between systems.^3^ For instance, the need for interoperability among various integrated systems, data communication standards and file formats can bring more complexity to the already existing complex healthcare system.^2,4^ Thus, new and unforeseen risks of HIT systems keep emerging, adding additional layers of healthcare complexity, which can cause patient inconvenience, service delays and even harm to patients.^5^

Various types of issues are associated with HIT systems. Some problems are technical, enfolding software or hardware design issues and human or organisational issues related to socio-technical contexts influencing the interface for human–computer interaction.^4^ Health information technology system software issues can be of different types, such as systems configuration (problems with default settings), software accessibility and availability^6^ and software functionality (poor user interfaces, fragmented displays).^7^ Consequences of these problems can range from inconvenience and workflow interruptions to patient harm, affecting whole systems, multiple facilities and sometimes even the entire healthcare organisation.^8^

Healthcare quality improvement and safety have always been the ideal—continuous improvement means ‘every defect should lead to improvement’.^9^ Throughout time, the collection of incident reports has become one of the essential safety management tools in the healthcare system. The incident reports are in the form of free-text narratives, i.e., a description reported by the healthcare professionals after things have gone wrong, and to recommend how to improve healthcare practice. The reporting process reflects on what happened, how it happened and what might have reduced or ameliorated the situation by capturing both near misses and adverse events.^10^ An accessible, valuable and usable incident reporting system functions as an information provider for continual safety improvement processes.^11^ Incident reporting, in particular, has the potential for specific problems to be identified and characterised, such as problems arising from unintended interactions between human and HIT systems in practice. Therefore, studying, examining and analysing healthcare incident reports provide a basis for improving healthcare quality and safety.

A classification system tailored to health information technology (HIT-CS) was developed by Magrabi et al.^8^ to identify, characterise and address the issues arising from HIT in healthcare. This classification adopted a ‘bottom-up’ approach for deconstructing incidents, categorising HIT-related issues and extracting meaningful information. Problems can be categorised based on human or technical-related issues, whereas technical problems can be divided into hardware and software-related issues. A single incident can be categorised into more than one incident type or characteristic using this framework.^8^ The HIT-CS also offers to identify the contributing factors and the consequences of the incidents that occurred. On the other hand, the inductive approach, i.e., thematic analysis, helps extract information that it may not be possible to extract using deductive analysis. In-depth knowledge can be derived to characterise and address HIT-related issues by combining these analysis techniques.^12^

In collaboration with the Swedish eHealth Agency and Swedish Data Inspection, the Swedish Medical Product Agency aims to deliver improved healthcare by disseminating an incident alert on medical devices. According to the stipulation, incidents related to national and medical information systems must also be reported to reflect a disparity between healthcare quality and patient safety. Healthcare providers from all regions in Sweden are strongly requested to improve process measurement and deliver leadership to reduce the risks associated with HIT systems by reporting relevant incidents.^13^

Much research has been conducted on HIT incidents^4,8,14–18^; however, no published articles were found that explored HIT-related issues in Sweden. A recent systematic review on problems with HIT indicated that 29 of 34 articles originated from the USA, the UK and Australia.^19^ These studies focused on a general review of a set of incidents collected from the incident database or through various other means. Nevertheless, more research needs to be conducted, focusing mainly on in-depth analysis of software-related HIT incidents. Therefore, there is a need for qualitative, both deductive and inductive analyses to explore the software-related incidents that occur in routine clinical practice in Swedish healthcare.

As we study healthcare settings and complex HIT systems that involve multiple actors, a systemic view can help us understand the interconnected HIT systems, components, modules and technologies used to make the entire healthcare organisation work.^20^ It is not enough to understand the underlying mechanism and emergent dynamics of the HIT systems, but rather it is necessary to understand how and why things went wrong in Swedish healthcare to inform future preventive and corrective strategies. By recognising different perspectives, we introduce a crucial aspect of an existing classification system, i.e., HIT-CS, combined with the inductive approach, i.e., thematic analysis. The study focuses on exploring HIT system-related software issues (such as software functionality and system configuration) through the lens of HIT-CS and thematic analysis as part of healthcare quality improvement activities. The study aims to examine the types of software problems posing risks to healthcare quality and patient safety.

## Methods

This is an exploratory, desktop analysis-based qualitative study. Data was collected from an organisation responsible for the incident repository, which was then analysed using both deductive and inductive approaches.

### Data collection

A total of 46 HIT incident reports were delivered by one of Sweden's national incident reporting repositories. The incidents were collected in two different time periods, and the two sets of incidents were later combined for data analysis. The organisation in charge of the incident repository has been represented as the national authority responsible for the regulation and surveillance of the development, manufacturing and sale of pharmaceutical and medical products. This national authority has been tasked with improving healthcare quality and patient safety by implementing digital incident reporting systems with the help of the corresponding county council. The organisation is also well known for spreading the knowledge about pharmaceutical and medical products, strengthening the knowledge of sustainable manufacturing and promoting the development and use of criteria for environmental considerations.

The total sample (*n* = 46) of the software-related incident reports, i.e., free-text narratives, ranged from June 2019 to December 2021 and was reported by healthcare professionals from all the regions in Sweden. These incidents were filtered and separated from the general set of medical device-related incidents before the delivery. Reports that contained identifying personal and sensitive information were partially edited by the data provider to de-identify them. The reports were delivered in Swedish and then translated into English by a linguistic specialist with expertise in both Swedish and English.

A follow-up communication was made by the principal investigator to extract additional information and to have a better understanding of the context of the things going wrong. However, no extra detail was obtained due to the absence of a response from the data provider; and we were informed that the administration was undergoing internal re-organisation due to the ongoing pandemic.

### Data analysis

**Figure 1.**
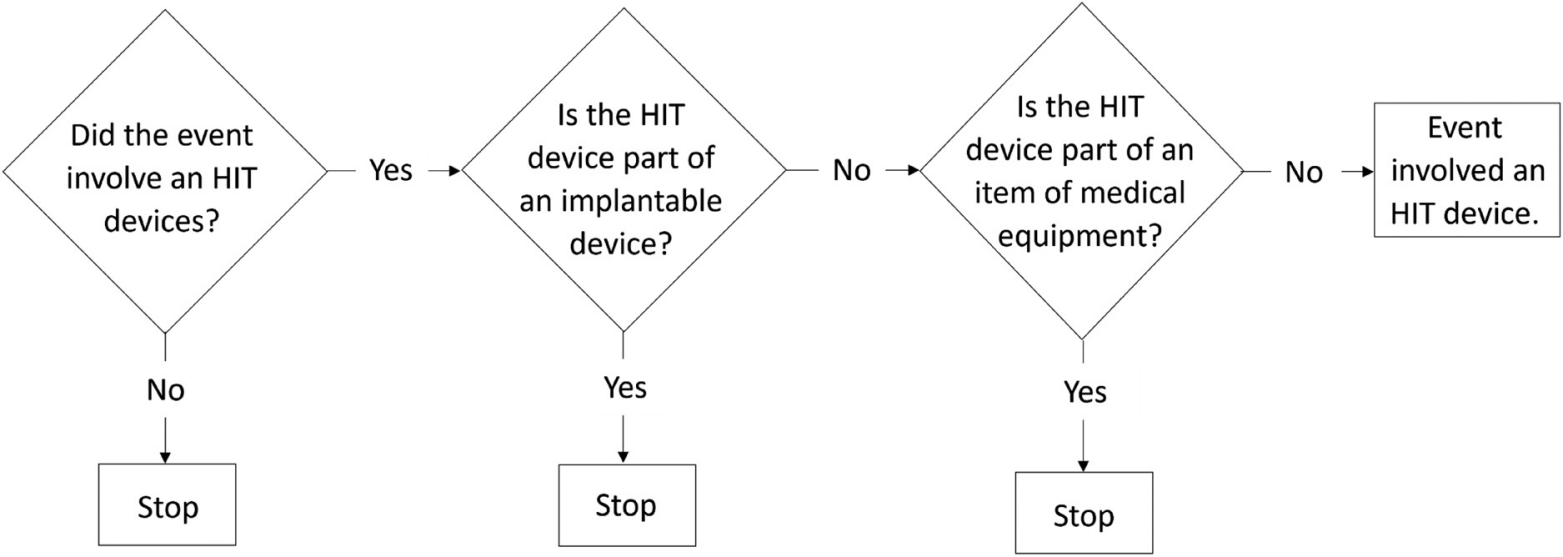
The algorithm for identifying HIT-related events adapted from The Joint Commission.^21^

Identifying the software-related incidents involved a two-step process. The total sample of the incidents was first subjected to an HIT algorithm^15^ for initial identification of the HIT incidents. The inclusion criteria involved the incidents being only associated with an HIT system rather than a part of medical equipment or implantable device (see Figure 1).^21^

After applying the HIT algorithm, the included incidents were then analysed using an existing classification system proposed by Magrabi et al.,^8^ namely the HIT-CS, to identify the software-related incidents. The proposed framework was used to determine the type of software issue and the type of consequence of the incident. The incidents containing at least one software issue were included since more than one category was involved in some incidents. Other categories determined, such as use-related, machine-related and hardware-related problems, are beyond the scope of this article.

The combination of two information fields of the incident reports, i.e., ‘event description’ and ‘manufacturer's conclusion’, was considered rather than each separate field for the application of the existing framework. This is because there were some variations in the details between these two information fields. Also, considering each field for data classification would have resulted in different results, which is beyond the scope of the study.

The incidents associated with software issues were then subjected to thematic analysis proposed by Braun and Clarke.^22^ The themes were extracted and presented under the category assigned by the HIT-CS. The thematic analysis was also used to extract information on actions taken to mitigate the risks, particularly from the field of ‘manufacturers’ measures’. The application of the HIT-CS and the generation of the themes were performed and regulated on a semantic level. The explicit content of the incident reports was examined without making any assumptions about the latent underpinnings of the data.

Both deductive (HIT-CS) and inductive (thematic) analyses were performed by the principal investigator and were cross-checked by the second coder. Inter-rater reliability was achieved for the HIT-CS incident category using the Kappa score calculation. The software-related incidents included were considered for inter-rater reliability, i.e., the Kappa score calculation. Both the coders reached a harmonious consensus if there was any difference of opinions on the thematic analysis.

## Results

Of 46 incident reports collected, 45 were included, and only one was excluded using the HIT algorithm. The 45 incidents included were subjected to the HIT-CS to identify the software-related incidents. A total of 31 software-related incidents were identified, which were then analysed thematically.

**Figure 2.**
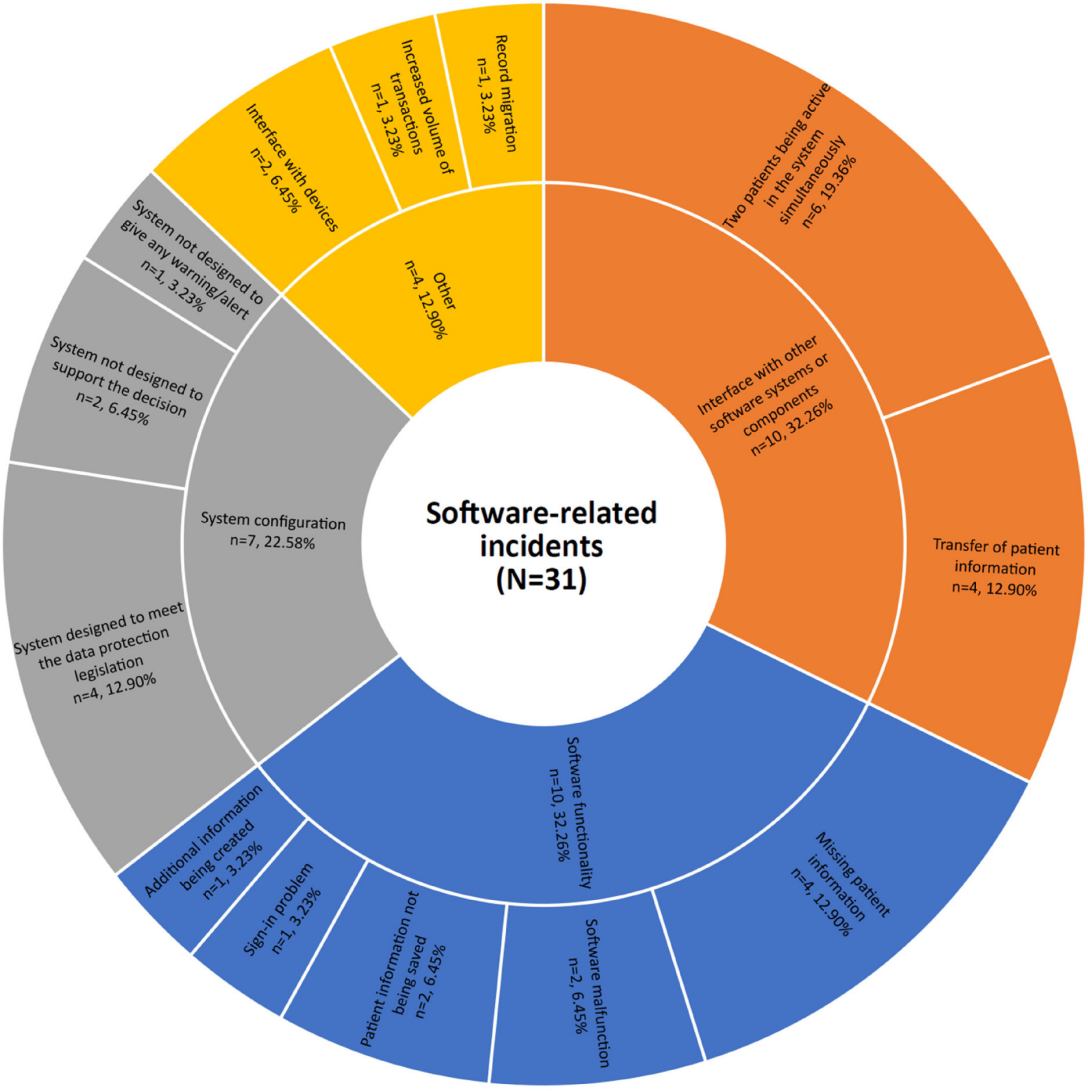
Software-related issues categorised using the HIT-CS and thematic analysis.

Of these 31 incidents, six types of issues were identified using the HIT-CS. The types of software problems included software functionality (*n* = 10), interface with other software systems or components (*n* = 10), system configuration (*n* = 7), interface with devices (*n* = 2), record migration (*n* = 1) and increased volume of the transaction (*n* = 1). These issues were further grouped into various themes, presented under the rubric of each software-related issue (see Figure 2).

The deductive analysis of these incidents was considered for inter-rater reliability, i.e., the Kappa score calculation. Inter-rater reliability between the coders was κ = 0.88 (*p* < 0.001, 95% CI 0.82–0.95).

A short illustration of each incident and its relevant type of issue, consequences and action taken is presented in Table 1.

**Table 1.** Short illustrations of each incident comprising the type of issue, consequence and action taken to reduce the risk.

IR no.	Short description of the core issue	Type of software issue	Consequence	Action taken to reduce the risks
IR_1_	Incorrect information in the section of ‘concluding summary’ of the patient record was sent electronically due to problems with the message threading, i.e. two messages entered the multilingual software platform simultaneously from each thread.	Interface with other software systems or components	An arrested or interrupted sequence or a near miss	The current part of the system was re-configured
IR_2_	An error due to a current software bug occurred when the user double-clicked on two different patients belonging to different value units while navigating from the occupancy list menu option, i.e. ‘distribution check’ to ‘distribution list’. This resulted in displaying two tabs with care documentation with two patient information creating a risk of confusion.	Interface with other software systems or components	An arrested or interrupted sequence or a near miss	The software bug was fixed
IR_3_	Three patients’ reports were missing because the system's validation of entered dates and times forced the user to end the operation on a date later than the present day.	System configuration	Harm to a patient or an adverse event	A software update was performed
IR_4_	A previously identified software issue caused the intervention type ‘single intervention’ not to be marked as ‘completed’ in the patient record even after it was marked ‘completed’ by the nurse.	Software functionality	An incident with a noticeable consequence but no patient harm	The error in the software was corrected
IR_5_	The patient shown in the Care Portal is not the same as the active patient in the Care Documentation due to an error in the program code.	Interface with other software systems or components	An arrested or interrupted sequence or a near miss	A delay was added to the program code
IR_6_	It was possible to have two patients active at the same time in the flow of ‘monitoring patient’ and ‘record overview’ since the desktop did not sense the patient change.	Interface with other software systems or components	An arrested or interrupted sequence or a near miss	Handling of mouse events was added
IR_7_	Incorrect patient data in the public dental system due to incorrect graphic codes delivered from another region.	Record migration	An incident with a noticeable consequence but no patient harm	The program codes were updated
IR_8_	A software error led to a problem in creating a new treatment template from a previous template.	Software functionality	An incident with no noticeable consequence	A hotfix was developed to update the program
IR_9_	A wrong script run in the region incorrectly deleted multiple prescriptions that had ‘change of clinic’.	Software functionality	An incident with a noticeable consequence but no patient harm	The script was corrected
IR_10_	A software bug created an error message that saving the waiting list items was impossible.	Software functionality	An incident with a noticeable consequence but no patient harm	The software was upgraded with a bug fix
IR_11_	A delay in transferring messages from one system to another due to the memory problem suffered by one of the systems.	Interface with other software systems or components	An incident with a noticeable consequence but no patient harm	The service was restarted to clear the message queues automatically
IR_12_	An error in the code prevented the text, i.e. ‘Terms of Benefits’ for the elderly, from displaying.	Software functionality	An incident with a noticeable consequence but no patient harm	The software code was upgraded with the bug fix
IR_13_	A software error allowed the staff to sign in to the HIT system without the required competence.	Software functionality	A hazardous event or circumstance	The correction consisted of changing the system
IR_14_	Certain information about students’ health record was thought to be missing; however, student record connected to the HIT system was designed to meet the data protection legislation and the Patient Data Act.	System configuration	An incident with no noticeable consequence	A report was sent to the Data Inspectorate
IR_15_	Information about students’ guardians was thought to be missing; however, student record connected to the HIT system was designed to meet the data protection legislation's requirements for privacy.	System configuration	An incident with no noticeable consequence	The risk of unauthorised access was assessed
IR_16_	An algorithm for generating patients’ IDs changed due to the need for a new module in the system resulting in multiple prescriptions not being transferred.	Interface with other software systems or components	An incident with a noticeable consequence but no patient harm	The algorithm was corrected by the developer
IR_17_	The wrong integration of two different systems allowed a browser to simultaneously display two patients’ information on the screen.	Interface with other software systems or components	An arrested or interrupted sequence or a near miss	Users were informed of the important safety notice
IR_18_	A software error did not allow the patient to change in Care Portal while keeping another patient in the Care Documentation, creating a risk of confusion.	Interface with other software systems or components	An arrested or interrupted sequence or a near miss	Necessary design changes were made to eliminate error
IR_19_	Some texts that the care staff has entered and saved in the patient record become inaccessible.	Software functionality	A hazardous event or circumstance	A review of the vigilance investigation was performed
IR_20_	A conversion program from a HIT system stopped the vaccination files not being transferred to the National Vaccination Register.	Interface with other software systems or components	An incident with a noticeable consequence but no patient harm	An extension of test protocols was added to solve the issue
IR_21_	A software error caused the administered dose to no longer be visible in the dose administration list.	Software functionality	A hazardous event or circumstance	A software error was detected during regression testing and corrected
IR_22_	An error in the program code led to two different patients ending up in the same context, in which the affected modules are Care Administration, Care Documentation and Care Portal.	Interface with other software systems or components	An arrested or interrupted sequence or a near miss	The program code was corrected
IR_23_	Medicines with expired prescriptions (validity period) were not shown during a care session, which was thought to be missing prescriptions from the system.	System configuration	Harm to a patient or an adverse event	Impact assessment and appropriate measures were recommended
IR_24_	The number of beds available in the regional server exceeded, and the system was not designed to give any warning.	System configuration	Harm to a patient or an adverse event	The system was functionally tested and solved
IR_25_	Information about students’ relatives with protected ID was thought to be missing; however, student record connected to the HIT system was designed to meet the data protection legislation and the Patient Data Act.	System configuration	An incident with no noticeable consequence	The risk of unauthorised access was assessed
IR_26_	The dose saved in the LINAC did not correspond to what was registered initially due to an interface issue.	Interface with devices	An incident with no noticeable consequence	The event was considered a safety issue
IR_27_	A digital health record system imported from another municipality did not reveal certain patient information to meet the data protection legislation act, which was thought to be missing information from the system.	System configuration	An incident with no noticeable consequence	A formal report was sent to the relevant party
IR_28_	A new computer in the radiology department stopped working due to a software malfunction.	Software functionality	An incident with a noticeable consequence but no patient harm	The software problem was corrected
IR_29_	If the X-ray first sends answers to one examination code and a moment later sends answers to the other code, the X-ray referral system can incorrectly add one of the answers to the history.	Interface with devices	An incident with a noticeable consequence but no patient harm	The logic in the program code was changed to fix the issue
IR_30_	The system could not handle the processing of a large number of prescriptions (e.g. more than 100 prescriptions) for a limited period due to a software issue.	Increased volume of transactions	An incident with a noticeable consequence but no patient harm	The software issue was solved using a rigorous testing protocol
IR_31_	The alarm log showed two different distributed alarm systems (components), resulting in no triggering or generating of an alarm (software malfunction).	Software functionality	An incident with no noticeable consequence	The risk was analysed and found to be under acceptable cumulative risk

### Software functionality

From 10 software functionality-related problems (IR_4,8,9,10,12,13,19,21,28,31_), five themes were formed. These themes were ‘missing patient information’ (*n* = 4), ‘patient information not being saved’ (*n* = 2), ‘software malfunction’ (*n* = 2), ‘additional information being created’ (*n* = 1) and ‘sign-in problem’ (*n* = 1).

#### Missing patient information

Of software functionality-related issues, four incidents were grouped under this theme (IR_9,12,19,21_). Certain software functions caused patient information to be missing from the system. For example, a planned change to the drug module was made to the current version, preventing the text, i.e., ‘terms of benefit’ for elderly patients, from being displayed.

Two of these incidents fell under the HIT-CS category of ‘an incident with a noticeable consequence but no patient harm’ (IR_9,12_), and the other two under ‘a hazardous event or circumstance’ (IR_19,21_). The actions taken to solve these issues were correcting the program script and upgrading the software code with the bug fix.

#### Patient information not being saved

A couple of incidents (IR_4,10_) were identified, which did not allow the ‘save’ function to save the information entered by the care staff. For instance, an error manifested itself after upgrading to a new version of the system, permitting an error message to pop up, namely ‘not possible to save’ for the waiting list item with an attached document.

These incidents were classified as ‘an incident with a noticeable consequence but no patient harm’ using the HIT-CS. In both cases, the error in the software was corrected, including the bug fix.

#### Software malfunction

Two incidents were determined within this theme (IR_28,31_), causing the system not to work properly. For example, a computer in radiology stopped functioning due to software problems.

One of these incidents resulted in a noticeable consequence, i.e., delay in care delivery, and another with no apparent consequence. One of the problems was solved by correcting the software, and the other did not require any rectification as the risk was considered acceptable.

#### Additional information being created

Only one theme was generated under this theme (IR_8_). The software functionality issue led to the production of additional templates; for example, a decision-support system automatically began to create a new treatment template from the previous ones.

The incident had no noticeable consequence; however, the issue was solved by developing a ‘hotfix’ for updating the program.

#### Sign-in problem

One incident was found to be incorporated with a ‘sign-in issue’ (IR_13_). For example, a software error allowed the staff to sign into the system verifying the user's permission to sign against the current process, but not the required competence.

The incident could potentially lead to adverse events or near misses: however, the system error was corrected by changing the system.

### Interface with other software systems or components

From 10 software interface-related identified problems (IR_1,2,5,6,11,16,17,18,20,22_), two themes were generated, namely ‘two patients being active in the system simultaneously’ (*n* = 6) and ‘transfer of patient information’ (*n* = 4).

#### Two patients being active in the system simultaneously

Six incidents were identified under this theme (IR_2,5,6,17,18,22_). A common concern was noticed when two different patients were active simultaneously in two different components or modules of the same system.

One of the examples of such software interface-related problems was regarding context management when navigating from the occupancy list menu option ‘distribution check’ to ‘distribution list’. The error occurred in the flow when the user double-clicked on two patients belonging to different value units. The error meant two tabs with care documentation were displayed, one from each care unit. In one care documentation, the patient had not been activated, but there was an incorrect previous patient with a risk of confusion regarding patients.

These incidents resulted in ‘an arrested or interrupted sequence or a near miss’, according to the HIT-CS. These incidents did not reach the patients; however, there were risks of staff confusion, and medical decisions could have been made on incorrect grounds. Actions taken to solve the problems were based on the presence of software errors, for example, fixing the program code.

#### Transfer of patient information

Four incidents were grouped under this theme (IR_1,11,16,20_). Of these, two incidents comprised ‘no transfer’ (IR_16,20_), one consisted of ‘incorrect transfer’ (IR_1_), and another was caused by ‘delayed transfer’ (IR_11_) of patient information.

For instance, an interface issue occurred between an HIT system used in primary, child and school healthcare, responsible for communicating different types of care documents such as prescriptions, referrals and certificates, and a system incorporated into the National Vaccination Register. Communication software stopped the traffic due to an error in the conversion program, which was supposed to convert the vaccination data into a required format supported by the National Vaccination Register. This means that some other traffic was turned upside down and queued up, causing files not to be transferred.

Three of these incidents comprising ‘no’ and ‘delayed’ transfer of patient information resulted in ‘an incident with a noticeable consequence but no patient harm’, and the other in ‘an arrested or interrupted sequence or a near miss’. Several measures were taken to resolve the issues, for example, reconfiguring the system, restarting the service and correcting the program algorithm.

### System configuration

From seven system configuration issues (IR_3,14,15,23,24,25,27_), three themes were found. These themes included ‘system designed to meet the data protection legislation’ (*n* = 4), ‘system not designed to support the decision’ (*n* = 2) and ‘system not designed to give any warning/alert’ (*n* = 1).

#### System designed to meet the data protection legislation

Of the system configuration-related issues, four incidents were categorised under this theme (IR_14,15,25,27_). These incidents were reported based on the reporters’ perceptions regarding certain system functions intended to protect individual information based on data protection legislation.

For example, a medical record system, among other things, was used primarily in primary care and school health care—different types of care documents, such as prescriptions, referrals and certificates. Students’ personal information and information about class affiliation were regularly loaded into the medical record system from various student administrative systems. The student's guardian was also entered as a relative with contact information. A function in the system, i.e., ‘clear guardian before student import’, was set up to ensure that no marking for a person who was no longer a guardian remained and no disclosure of information to any unauthorised persons took place. However, the perception of the function ‘clear guardian before student import’ by the user or care provider was that all information about relatives marked as guardians should have been deleted. This was not the case; rather, it was only the tick for guardians for those who were removed. In practice, deleting the entire record would mean that all personal information about the parent who ceased to be a guardian would have disappeared from the system. This would then mean that personal data was processed without legal support, which was inappropriate and incompatible with data protection legislation and the ‘Patient Data Act'.

All of these incidents resulted in ‘an incident with no noticeable consequence’ in accordance with the HIT-CS. These incidents were not considered reportable cases because of the reporters’ misunderstanding and lack of awareness about the intended function of the system. Among several actions taken to reduce the risks was the assessment of the risks of unauthorised access to patient data and the sending of a formal incident notification to the Data Inspectorate.

#### System not designed to support the decision

Of these configuration issues, two incidents were reported as being associated with the system, which was not designed to support the decision of the care delivery (IR_3,23_). As an illustration, there was an error in the product, which did not allow a given care intervention to be performed on the day it was supposed to. The care delivery was added for performance at a later date because the system's validation of entered dates and times forced the user to complete the performance on a date later than the present day.

These incidents posed severe risks to patient safety and were categorised as ‘harm to a patient or an adverse event’. Impact assessment and appropriate measures were recommended, including the software update to meet the required system criteria.

#### System not designed to give any warning/alert

One incident was identified for which the system needed to be designed to give a warning in case of exceeding the number of beds in the county-wide server (IR_24_). Consequently, the system alarm did not mediate through the staff's alarm for the care event.

The incident posed the highest risks of causing ‘harm to a patient or an adverse event’, according to the HIT-CS. The error was recreated by several other customers and vendors and escalated with maximum severity and priority in both local administration and at the level of vendors.

### Interface with devices

Two incidents were identified under this category; no particular theme was generated (IR_26,29_). For instance, the interface with the Medical Linear Accelerator (LINAC) was not working. The dose saved in the LINAC did not correspond to what was registered. Since it was not a delivery issue but a registration problem, it did not render a wrong clinical decision.

Of these incidents, one (IR_26_) was not considered a safety issue, and no action was taken. However, the other (IR_28_) caused significant frustration to the healthcare professionals and delays in patient diagnosis. The corrective measures were taken by extending the enforcement conditions for incoming X-ray response, i.e., changing the logic in the program code.

### Record migration

A single incident was associated with record migration (IR_7_). For example, patient data, such as tooth graphics and missing teeth from the public dental system delivered from another region, in some cases, needed to be corrected.

The incident could lead to a severe deterioration of a patient's health; however, in this instance, it only caused some delays in the care delivery. Hence it was categorised as ‘an incident with a noticeable consequence but no patient harm using the HIT-CS’. The problem was solved by updating the program codes in the software.

### Increased volume of transactions

Only one incident was determined to be an ‘increased volume of transactions’ (IR_30_). The incident involved a blockage of prescription transfer from one system to another due to its large volume.

The incident caused a significant delay to its regular operation until a local approach was considered as a solution, i.e., transferring the prescriptions multiple times in small batches. The problem was resolved using a rigorous software testing protocol.

## Discussion

Swedish society has prioritised digital transformation, especially as it relates to healthcare. However, adopting and implementing new HIT systems could seriously impair system interfaces and interoperability. Healthcare is a complex socio-technical system, and some hold the opinion that information technology can cause errors rather than fix them.^23,24^ Moreover, the challenges faced can depend on the type of HIT systems and software embedded in them, and thus each problem becomes unique to resolve. For example, one has to look through the underlying mechanisms of the specific HIT system, such as Electronic Health Records (EHRs) or Health Information Systems and how they are interconnected or interfaced with other systems, such as Radiology Information Systems and Picture Archiving and Communication System.^12,14^ Very little research has been done on in-depth analysis of HIT incidents connected to the software. The present study provides insight into software-related challenges in Swedish healthcare through the lens of incident reports. A list of software deficiencies was identified: software functionality, interface with other software systems or components, system configuration, interface with devices, record migration and increased volume of transactions.

Prior studies pointed out that software issues with medical devices are common and can potentially negatively impact patient care.^25,26^ The current study found that software interface issues encompassed 37% of the identified software-related incidents. Working in a complex socio-technical system, healthcare professionals had to operate a range of HIT systems in their clinical practice, and the parallel utilisation of different non-integrated components/modules confused the context management. This complexity further increased the risk of conflicting information, contributing to frustration among healthcare professionals and potentially leading to patient harm.

System software used within healthcare was mostly independently developed^27^; consequently, the exchange of patient information across healthcare systems has yet to be seamless or efficient.^15^ The decentralised nature of regional healthcare added another layer of complexity to the information transfer within Swedish healthcare. The results of this study indicate that the patient information is frequently in text form, which is non-standard, non-structured and uncoded, making the transferring of information difficult,^24,28^ and the patient data from local systems had to undergo format transformation to be accepted by the national system. The conversion process escalated the complexity of the healthcare system and increased the risk of interface issues.^24,29^

The interface issues also manifested in software–user interaction. The current study found that the users did not perceive specific software configurations in the designated way. The perceptual gaps between software users and developers were also spotted in Ndabu et al.'s^30^ work, which indicated that IT professionals do not fully understand the clinical setting and how workloads and other patient-related variables affect the use of software. As a result, software developers were not able to design a robust system that aligned with the clinical workflow, and users were frustrated about the disrupted care process.

With a range of HIT systems, although the manifestations of these issues were obvious, such as the system not being designed to support the decision, the underlying mechanisms were almost obscure. This is a hallmark of complex systems with the fact that different systems from different vendors are used in healthcare, and based on each system and end-user, different contributing factors may cause the problems that arise.^4,15^ The exact mechanisms underneath software-related incidents still need to be clarified, provided that the interpretation of the result is quite general. The system fixes or upgrades may lead to further issues, and it might be more challenging to identify and address the mechanisms underlying those issues. Jabin et al.^12^ argued in their study that the technical problems at the system level might be apparent in any pathway of the clinical workflow resulting in patient harm that requires system-level solutions. Potential local solutions should be managed so that early detection and mitigation play a vital role. This means that the systems must be designed to prevent specific errors. Considerable thought and ingenuity will need to be supported and validated by studies, including observational and ethnographic studies. This approach will prevent and mitigate errors in the context of different clinical workflow steps in which they occur.^12^

### Implications for practice

In our previous studies on HIT incidents, we devised a general set of preventive and corrective strategies since most of the incidents were contributed by human factors than technical factors.^18,24,26^ Such strategies were developed to mitigate the risks associated with either design and developmental challenges, implementation and use, or monitoring, evaluation and optimisation. In this study, we present some of the local solutions that a medical engineer or HIT technician may consider in case of any specific issue as described. When investigating a problem, a check should always be made to determine whether similar errors have occurred before. This should be done by identifying modules/components relevant to the problem and reviewing previous cases. Caution must be taken since each HIT-related system problem is unique, and therefore, each may require root cause analyses supplemented by a mechanism for collecting additional information from other medical sources, such as medico-legal files and complaints. While an investigation is underway to find the root cause of the problem, meetings with all affected customers or vendors with the same version of the software should be planned and scheduled. This meeting should be followed up with a review of the vigilance investigation and the security announcement, which will then be distributed to the end users.^4,15,31^

#### Establish clear patient context in an interfaced healthcare environment

No matter how comprehensive a single product's features are, there will always be new HIT functionality and stand-alone software developed that need to be interfaced with the current system. A poor interface among them could confuse patient context management, such as two patients being active simultaneously.^32,33^ A measure that can be taken to ensure that patient context is followed is adding a code for the ‘patient change’ function while handling mouse events that allow selecting a patient without triggering a patient change. Adding a ‘mouse handling event’ can also be another local HIT solution that allows selecting a patient without triggering a patient change when two patients are active simultaneously.

Other solutions may include entering a ‘delay’ in the program code so that two quick double-clicks on different lines in the Occupancy List cannot cause two patients to be active simultaneously on two tabs with care documentation. Additionally, a ‘risk-reducing measure’ may be adopted in the form of extra checks in the program code to ensure that the patient identities in both the tabs of Care Documentation are the same.

#### Ensure decisive information is displayed for decision-support interventions

This study outlined the software issues that supportive information was not provided in the form of an alert, which led to the neglecting of decisive information, such as the number of beds in the regional server exceeded, ending up with no warning. A potential solution to these issues could be adding an ‘alert’ in the program code so that decisive information cannot be neglected. Besides, it would be ideal that the alert has a clear call to action, concise text and a severity tier.^18,34^ Using standardised terminology and colour schemes could further increase users’ sensitivity to decisive information.

#### Perform ‘robust testing/regression protocol’ in system design prior to deployment

Whilst software problems can be progressively ‘designed out’, it would seem that in the meantime, the dire need for appropriate testing to avoid or mitigate the occurrence of these errors in the field, which may have a more significant impact on the patient, providers and the healthcare in general. With considerable thought and ingenuity, some of the software errors can be phased out by identifying them in advance during robust testing or regression protocol in the development phase prior to the deployment of the system. For example, the software issues associated with software functionality and system configuration identified in our study could have been avoided if regression protocol had been conducted beforehand.^35^

Resolving HIT-related issues can be expensive in most cases, but the need for more adoption of these systems makes it worse in terms of time and resources. Bardhan and Thouin^35^ demonstrated in their study significant implications on the cost-quality trade-off related to using the HIT system. This implies that the application of HIT systems may result in cost inflation and be counterproductive if the healthcare workflow process^18^ and different treatments^35^ (that work and those that do not) need to be properly understood.

#### Plan carefully for immediate local fixes and system transition

Risks associated with system issues, including system updates or upgrades, always exist in complex healthcare despite taking constant preventive measures.^18,36^ In case of issues in upgrading a system, before investigating a problem, a check should always be made of whether similar errors had occurred before the upgrade took place. This should be done by identifying modules or components relevant to the problem and reviewing previous cases. It is possible that a module or component might have been gripped with a software error, for example, an external bug. Therefore, an upgrade with the ‘bug fix’ should be performed to restore any relevant module or component of the systems.

#### Ensure configuration and communication between HIT systems

Configuration of different HIT systems or the components of these systems are essential so that they are adequately interoperable. These systems should communicate with each other to ensure access to prior medical examinations.^37,38^ For example, it is only sometimes possible that incorrect patient information from another care unit is sent from one patient information system to another due to human factors. The ‘processing step error’ may emerge when the message is translated from format A to format B, specifically in a program module that encodes text. The error may also occur due to multiple message threading problems, i.e., several message threads may be used against the Enterprise System platform. In this case, the current part of the system should be re-configured to use only one thread (in practice) to prevent collisions in the program module from occurring again. Deleting incorrect information manually from the patient record is also necessary in such cases.

Another example may involve the service on the communication platform, causing a delay in transferring messages. There may be a potential ‘memory leak’ in the service code or a shortage of memory on the communication platform, resulting in message traffic being stopped. In such a case, it is necessary to ‘restart’ the service on a regular basis. A routine for ‘automatic restart’ of the service (with manual monitoring) may be introduced/set up if not already in place to clear the queues in the message transfer that fall out as errors. This measure should be taken for all similarly affected installations by identifying similar software modules or components.

Additionally, the system can also be configured to operate the high volume of patient data transactions by setting the limit ‘high’ in the program code. This setting should be supplemented by supplying and adding the information of the maximum limit for ‘medical record transaction’ to the system protocol. This will eventually help healthcare professionals in understanding the system limitation in the transaction of patient records and mitigate the risks of unexpected events.

#### Provide training on general data protection regulation and information security awareness

Healthcare staff need to receive adequate paid time for professional training jointly organised by the healthcare organisation and HIT vendors.^26,39,40^ When users misunderstood and were unaware of the software's intended purpose, they did not regard some incidents as reportable cases, and thus certain issues remained. When providing ongoing training for HIT end users, the importance of compliant incident reports should be highlighted. In addition, healthcare should receive guidance about data protection legislation and security awareness of the HIT applications. Such training can eventually mitigate the software-related incidents that are contributed by users.^24^

Suppose certain information, such as data on a patient's relatives, is missing in the patient record. Instead of assuming that the data has manually been deleted or not sent by the operator, one should look for the possibility that the information is designed for privacy as a default to meet the requirements of data protection legislation. It is also possible that the information about the relative has been removed because the person is no longer a relative; therefore, disclosing information to unauthorised persons may violate current data protection legislation. Sometimes, the patient may also refuse to transfer all or part of his/her medical record; however, this information must be noted in the medical records content assessments. Under the above circumstances, the care provider should not be under the impression that all information about the relatives should be deleted. Instead, information about the relative's data, such as address and contact details, must therefore be changed manually.

The privacy concern for the ‘data legislation act’ can also be solved by introducing a two-factor authentication function into the system when disclosing a person with a protected identity is necessary. The required information can be extracted using the technique of authorisation and access control, where access can only be made through authorised personnel after two-factor authentication. This will prevent the risk of unauthorised disclosure of personal data.

### Strengths and limitations of the study

Although there are important discoveries revealed in this study, these results must be interpreted with caution. The data collected in this study were from one of Sweden's national incident reporting repositories, which focused on medical products implemented within Swedish healthcare. The themes were generated from detailed and descriptive texts on HIT incidents, but the sample size, i.e., the number of incident reports, was relatively small compared to previous studies. Despite the small sample, the narratives were rich in texts with the combination of two information fields of the incident reports, ‘event description’ and ‘manufacturer's conclusion’, which provided us a sufficient holistic view of what went wrong and how they went wrong in the routine clinical practice of Swedish healthcare. This, in turn, helped us to devise preventive and corrective strategies for specific software problems that occurred. Moreover, we collected incident reports that provided instructive information on heterogeneous systems, such as actions taken to handle software issues in e-prescribing systems, administration systems and decision-support systems.

Secondly, both deductive and inductive analyses were adopted in the current study, allowing investigators to extract more detailed information from the data compared to adopting only one of the analysis methods. A few strategies were adopted to mitigate the potential subjective bias in the analysis: (a) involving two investigators independently in the coding process for both deductive and inductive analysis; (b) both analysis methods were conducted repeatedly until the investigators reached an agreement; and (c) adopting a time gap between deductive and inductive analyses.

Thirdly, due to the decentralised administration of Swedish healthcare, our results must be interpreted cautiously. In Sweden, municipalities and regions have complete autonomy regarding HIT investment, use and management. For example, the incidents collected were reported by healthcare professionals from different regions in Sweden, but the origin of the incidents was not included or displayed in the incident reports. As a result, the current study gave a broad overview of software-related problems in Swedish healthcare. If results are to be more tightly tied to a particular region or software, filters like location or type of software should be added during data collection. Notwithstanding these limitations, the results can be considered as alerts to inform healthcare digitalisation in Sweden to be more effective and safer.

## Conclusion

This study provides some insights into types of software issues and relevant consequences derived through the lens of HIT incident reports occurring in day-to-day Swedish clinical practice. A set of local solutions were devised that a medical engineer or HIT technician may consider applying to some of the software challenges identified in this study. The proposed solutions will help overcome the present challenges encountered in Swedish healthcare in daily clinical practice. It follows that systematic study of such HIT incident reports associated with software challenges should be a routine part of clinical practice for all major HIT implementations.
